# Compliance With Protocols for Prevention of Neonatal Group B Streptococcal Sepsis: Practicalities and Limitations

**DOI:** 10.1155/S1064744903000012

**Published:** 2003

**Authors:** Gwendolyn L. Gilbert, Moira C. Hewitt, Catherine M. Turner, Stephen R. Leeder

**Affiliations:** 1 Centre for Infectious Diseases and Microbiology Institute of Clinical Pathology and Medical Research Westmead Hospital Darcy Road, Westmead, NSW 2145 Australia; 2 Department of Public Health and Community Medicine University of Sydney Westmead Hospital New South Wales Australia; 3 Master of Applied Epidemiology Programme National Centre for Epidemiology and Population Health Australian National University Canberra Australia

## Abstract

*Objective:* To compare two protocols for intrapartum antibiotic prophylaxis (IAP) against neonatal group B streptococcal (GBS) sepsis, with respect to staff compliance, in a prospective cohort study in the obstetric units of a community hospital (A) and a university teaching hospital (B).

*Methods:* Cohorts comprised about 500 women attending antenatal clinics at each hospital (total 1096). Women identified as GBS carriers at 26–32 weeks'gestation and those who had intrapartum clinical risk factors (CRF) were eligible for IAP. Compliance was defined as the proportion of women eligible for IAP who received it according to protocol–as determined by audit of case records–and compared between hospitals and according to indication.

*Results:* Overall, 39% of women were eligible for IAP. Indications were GBS carriage alone (21%), CRF alone (13% ) and both (5% ). Compliance was similar for GBS carriers at both hospitals: 78% at Hospital A and 76% at Hospital B. However, because of the poor predictive value of screening before 32 weeks, only 65%of intrapartum GBS carriers actually received IAP. For women with CRF only, compliance was significantly lower at Hospital B than Hospital A (56 vs. 75%;* p*= 0.03).

*Conclusions:* According to currently recommended protocols, about one-third of healthy women are eligible for intrapartum antibiotics to prevent neonatal GBS sepsis. In practice, antibiotics are often used inefficiently because of poor compliance with protocols and poor predictive values of selection criteria. Better implementation strategies should improve compliance, but GBS vaccines are needed to replace prophylactic antibiotic use, with its associated disadvantages.


Infect Dis Obstet Gynecol 2003;11:1–9

### Compliance with protocols for prevention of neonatal group B streptococcal sepsis: practicalities and limitations

**Gwendolyn L. Gilbert[1], Moira C. Hewitt[2,3], Catherine M. Turner[1] and Stephen R. Leeder[2]**

[1]*Centre for Infectious Diseases and Microbiology, Institute of Clinical Pathology and Medical Research and* [2]*Department of Public Health and Community Medicine, University of Sydney, Westmead Hospital, New South Wales, Australia*
[3]*Master of Applied Epidemiology Programme, National Centre for Epidemiology and Population Health, Australian National University, Canberra, Australia*

***Objective:*** To compare two protocols for intrapartum antibiotic prophylaxis (IAP) against neonatal group B streptococcal (GBS) sepsis, with respect to staff compliance, in a prospective cohort study in the obstetric units of a community hospital (A) and a university teaching hospital (B).
***Methods:*** Cohorts comprised about 500 women attending antenatal clinics at each hospital (total 1096). Women identified as GBS carriers at 26–32 weeks' gestation and those who had intrapartum clinical risk factors (CRF) were eligible for IAP. Compliance was defined as the proportion of women eligible for IAP who received it according to protocol – as determined by audit of case records – and compared between hospitals and according to indication.
***Results:*** Overall, 39% of women were eligible for IAP. Indications were GBS carriage alone (21%), CRF alone (13%) and both (5%). Compliance was similar for GBS carriers at both hospitals: 78% at Hospital A and 76% at Hospital B. However, because of the poor predictive value of screening before 32 weeks, only 65% of intrapartum GBS carriers actually received IAP. For women with CRF only, compliance was significantly lower at Hospital B than Hospital A (56 vs. 75%; $p = 0.03$).
***Conclusions:*** According to currently recommended protocols, about one-third of healthy women are eligible for intrapartum antibiotics to prevent neonatal GBS sepsis. In practice, antibiotics are often used inefficiently because of poor compliance with protocols and poor predictive values of selection criteria. Better implementation strategies should improve compliance, but GBS vaccines are needed to replace prophylactic antibiotic use, with its associated disadvantages.

Key words: INTRAPARTUM ANTIBIOTIC PROPHYLAXIS; GROUP B STREPTOCOCCAL SEPSIS; COMPLIANCE

This study was funded by National Health and Medical Research Council Grant number 974089 – Screening pregnant women to prevent neonatal group B streptococcal sepsis. G. L. Gilbert, S. R. Leeder

Professor G. L. Gilbert, MD, Centre for Infectious Diseases and Microbiology, ICPMR, Westmead Hospital, Darcy Road, Westmead, NSW, Australia 2145. Email: lyng@jcpmr.wsahs.nsw.gov.au

© 2003 The Parthenon Publishing Group

### INTRODUCTION

Intrapartum antibiotic prophylaxis (LAP) can reduce vertical transmission of group B streptococcus (GBS) and prevent most cases of neonatal GBS sepsis[1]. However, the best way to identify women whose infants are at risk is controversial. When this study was planned, routine antenatal screening at 28–32 weeks' gestation and IAP for all carriers was established in a few Australian obstetric hospitals[2–4]. An alternative strategy based on clinical risk factors (CRF) during labor had been proposed[5] but not evaluated. Subsequently, consensus guidelines for prevention of neonatal GBS sepsis were published in the USA[6], but have been implemented inconsistently elsewhere[7,8]. The effectiveness of any strategy depends on compliance with protocols. Previous studies have shown variation in rates of compliance with IAP protocols of 65–85%, but there has been no direct comparison of compliance with different protocols[9–11]. The aim of this study was to compare two IAP protocols, in a community hospital (A) and a university teaching hospital (B), to assess compliance and factors affecting it.

### METHODS

### Study design

On the basis of a theoretical comparison of four strategies[5], we chose, for comparison, the two that had been judged to provide the best balance between the proportions of preventable cases of neonatal GBS sepsis and of women given antibiotics. We believed that randomization of strategies would be impractical and inclusion of an untreated control group unethical. Instead, we implemented both strategies, concurrently, and compared them. The two strategies were:

(A) IAP for all women identified as GBS carriers by antenatal screening at 26–32 weeks' gestation; and

(B) IAP for all women in whom one or more CRF was present on admission to the delivery suite or developed at any time up to 30 minutes before anticipated delivery. CRF for perinatal sepsis were defined as follows: a previous infant with GBS sepsis or GBS urinary tract infection; preterm labour (< 37 weeks' gestation); prolonged rupture of membranes (PROM, > 18 hours); intrapartum fever (see below).

IAP consisted of ampicillin, 1 g intravenously every 6 hours (or erythromycin 500 mg intravenously every 6 hours for women with suspected penicillin allergy) until delivery.

The study plan was to recruit and follow to delivery, cohorts of about 500 women at each of two hospitals in western Sydney. Cohort sizes were determined on the basis of estimated rates of GBS carriage and risk factors and to achieve confidence intervals within ± 5%.

### Hospitals and protocols

The obstetric unit at Hospital A delivers approximately 2600 babies per annum and has a special care (level 2) nursery. An IAP protocol based on strategy B was implemented in 1996, with in-service training of staff. The definition of maternal fever was a temperature > 37.5°C. Results of audits of patient records, conducted before and after the cohort study to assess compliance with this risk-factor-based protocol, have been reported separately[12].

A cohort of approximately 500 women was recruited at Hospital A during the first 6 months of 1997. Antenatal clinic staff approached consecutive women at their first antenatal visits, explained the nature of the study and asked them to participate. Demographic data from those who agreed were recorded immediately, and vaginal and anal swabs were collected at 26–32 weeks' gestation and repeated during labor. Women in the cohort were eligible for IAP if they had been identified as GBS carriers, and/or if one or more CRF was present on admission to the delivery suite or developed at any time until 30 minutes before delivery. Research nurses were responsible for in-service training of antenatal clinic and labor ward staff. Otherwise, protocol implementation was the responsibility of hospital staff.

Hospital B is a tertiary referral centre with an obstetric unit that manages approximately 4300 deliveries per annum; the hospital has both neonatal intensive care (level 3) and special care nurseries. The study protocol was generally similar to that at Hospital A except that a CRF-based protocol was introduced in early 1998, without specific in-service staff training; according to this protocol, maternal fever was defined as a temperature of 38°C. As for Hospital A, audits of patient records were done before and after recruitment of the study cohort to assess compliance with the risk-factor-based protocol alone[12].

Recruitment of the cohort occurred during an 8-month period in 1998–9. Research staff recruited women and, consecutively, obtained informed consent and collected demographic data and swabs from those who agreed to participate, at the same visit (at 26–32 weeks' gestation). Eligibility criteria for IAP at Hospital B were the same as for Hospital A except that maternal fever was defined as 38°C, according to protocol.

Compliance with delivery suite protocols for each hospital was assessed by review of medical records and defined as the proportion of women fulfilling the criteria for IAP, according to the corresponding protocol, who were given at least one dose of an appropriate antibiotic at least 30 minutes before delivery.

### Microbiology

Cultures from both hospitals were processed at the same laboratory. Full details of microbiological methods and results have been reported separately[13]. Briefly, all swabs were plated onto horse blood agar and GBS selective media directly and then placed in enrichment broth for overnight incubation before subculture onto agar. GBS carriers were defined as women from whom GBS was isolated from either vaginal or anal swab or both, by direct plating and/or after overnight enrichment culture. The medical records of GBS carriers were marked with a prominent coloured sticker.

### Neonatal outcome

Infants admitted to special or intensive care nurseries, with a diagnosis of suspected sepsis, were classified, after record review, as having:

(i) no sepsis;

(ii) clinical sepsis (negative cultures but clinical, radiological and/or other laboratory evidence of sepsis; response to antibiotic therapy); or

(iii) bacteriologically confirmed sepsis (significant isolate from blood or other sterile site culture).

### Statistical analysis

Data were entered into a Microsoft Access database and converted to an SAS dataset for analysis using SAS version 6.12 for Windows and Epi Info version 6.04b. Pearson's $\chi^2$ test or Fisher's exact test where used, as appropriate, to compare non-parametric data. Positive and negative predictive (PPV, NPV) values and confidence intervals (CI) were calculated in Epi Info.

### Ethical approval

Ethical approval for the study was granted by the Western Sydney Area Health Service Human Research Ethics Committee.

### RESULTS

GBS carriage rates and CRF are summarized in Table 1. The two cohorts comprised 1096 women who had full sets of antenatal and intrapartum GBS culture results. Antenatal and intrapartum GBS carriage rates were similar (27 and 24%, respectively) and did not differ significantly between hospitals. The PPV and NPV of antenatal screening for intrapartum GBS carriage were 69 (95% CI, 64–74) and 92% (95% CI 90–94), respectively. One or more CRF were identified during labor in 18% of women, most of whom (81%; 95% CI 75–86) had only one. CRF were present in similar proportions of GBS carriers and non-carriers.

### Indications for and compliance with protocols for IAP

According to labor ward protocols, 429 of 1096 (39%; 95% CI, 36–42) women were eligible for IAP (Table 2). Labor ward records showed that 361 women were given antibiotics during labor; 47 were given antibiotics for another indication

**Table 1** Indications for intrapartum antibiotic prophylaxis: antenatal and intrapartum GBS carriage rates and clinical risk factors

| | *Hospital A:* n = 581[3] n (%; 95% CI) | *Hospital B:* n = 515[3] n (%; 95% CI) | *Total:* n = 1096 n (%; 95% CI) |
|---|---|---|---|
| Antenatal GBS culture positive[1] | 151 (26; 22–30) | 140 (27; 23–31) | 291 (27; 24–29) |
| Intrapartum GBS culture positive[1] | 148 (25.5; 22–29) | 120 (23; 20–27) | 268 (24; 22–27) |
| History of GBS-infected infant[2] | 0/355 | 2/265 (0.75) | 2/610 (0.033; 0.004–0.12) |
| GBS bacteriuria | 4 (0.7) | 3 (0.6) | 7 (0.064; 0.026–0.13) |
| Premature labour (< 37 weeks) | 27 (4.7) | 24 (4/7) | 51 (4.7; 3.5–6.1) |
| PROM (> 18 hours) | 61 (10.5) | 71 (14) | 132 (12; 10–14) |
| Intrapartum fever | 20/563 (3.6)[4] | 28/479 (5.8)[4] | 48/1042 (4.6; 3.4–6.1) |
| Any risk factor | 95 (16) | 105 (20) | 200 (18; 16–20.5) |
| Single risk factor | 79 (14) | 82 (15.5) | 161 (15; 13–17) |
| Two or more risk factors | 16 (2.8) | 23 (4.5) | 39 (3.6; 2.6–4.8) |
| Risk factors in | | | |
| IP GBS carriers | 25/148 (17) | 31/120 (26)[5] | 56/268 (21; 16–26) |
| IP GBS non-carriers | 70/433 (16) | 74/395 (19)[5] | 144/828 (17; 15–20) |

[1]Sensitivity, specificity, positive and negative predictive values of positive antenatal screening for intrapartum carriage were 76.5, 88.4, 69.3 and 92.1%, respectively; [2]multiparous women only; [3]there were no significant differences betwen proportions at Hospitals A and B; [4]the different definitions of fever used at each hospital (see text) were used for calculation of incidence of risk factors and compliance with intrapartum antibiotic prophylaxis; difference not significant; [5]differences between GBS carriers and non-carriers not significant;
IP, intrapartum; GBS, group B streptococcus; PROM, premature rupture of membranes

**Table 2** Indications for intrapartum antibiotic administration and compliance with labor ward protocols for intrapartum antibiotic prophylaxis

| | *Hospital A* | | *Hospital B* | |
|---|---|---|---|---|
| | *Eligible for IAP[1] (% of cohort)* | *Given IAP as per protocol (% compliance; 95% CI)[2,3]* | *Eligible for IAP[1] (% of cohort)* | *Given IAP as per protocol (% compliance; 95% CI)[2,3]* |
| All AN GBS carriage | 151 (26) | 118 (78; 72–85) | 138 (27) | 105 (76; 69–83) |
| AN GBS carriage alone | 123 (21) | 96 (78; 71–85) | 108 (21) | 78 (72; 64–81) |
| AN GBS carriage and RF | 28 (4.8) | 22 (79; 59–92) | 30 (5.9) | 27 (90; 74–98) |
| All RF | 95 (16) | 73 (77; 67–85) | 103 (20) | 68 (66; 57–75) |
| RF alone[4] | 67 (11.5) | 50 (75; 62.5–84.5)[4] | 73 (14) | 41 (56; 44–68)[4] |
| Total (all indications) | 218 (37.5) | 168 (77; 71.5–83) | 211 (42) | 146 (69; 63–75) |

[1]According to either of two labor ward protocols, using original definitions of maternal fever (see text for details). Two women at Hospital B were admitted to delivery suite less than 30 minutes before delivery were excluded; [2]see text for details of protocols and exclusions; [3]percentage of women eligible for IAP who received it according to protocol; [4]significant difference in compliance between Hospital A and Hospital B ($p = 0.03$). All other differences not significant; AN, antenatal; GBS, group B streptococcus; RF, risk factors; IAP, intrapartum antibiotic prophylaxis

(usually endocarditis prophylaxis) or the first IAP dose was given less than 30 minutes before delivery and were excluded from analysis. Thus, 314 women, or 73% (95% CI, 69–77) of those eligible – 77% at Hospital A and 69% at Hospital B – were given IAP according to one or both protocols (Table 2). Most patients had only one CRF. The only significant difference between hospitals was in women with a single CRF, who were not GBS carriers (Table 3). Compliance varied significantly according to individual CRF. Compliance was highest when the CRF was fever and lowest when it was preterm birth. It was higher for patients who had more than one CRF or were also GBS carriers than for patients with a single risk factor who were non-carriers. Two or more CRF, or CRF plus

**Table 3** Compliance with intrapartum antibiotic prophylaxis protocols at Hospitals A and B for women with various risk factors

| | *Hospital A* | | *Hospital B* | | *All* | |
|---|---|---|---|---|---|---|
| *Indication* | *Eligible for IAP*[1] | *Compliance*[2] *(%; 95% CI)* | *Eligible for IAP*[1] | *Compliance*[2] *(%; 95% CI)* | *Eligible for IAP*[1] | *Compliance (%)* |
| Preterm delivery only | 19 | 7 (37; 16–62) | 13 | 4 (31; 9–61) | 32 | 11 (34)[7] |
| PROM only | 45 | 37 (82; 68–92) | 49 | 27 (55; 40–69) | 94 | 64 (68)[7] |
| Maternal fever[3] only | 12 | 12 (100; 73.5–100) | 15 | 13 (87; 59.5–98) | 27 | 25 (93)[7] |
| GBS bacteriuria | 3 | 1 | 3 | 2 | 6 | 3 (50) |
| Any single RF[4] | 79 | 57 (72; 61–82) | 80 | 46 (57.5; 46–68.5) | 159 | 103 (65)[8] |
| AN GBS carriers | 25 | 20 (80; 59–93) | 25 | 22 (88; 69–97.5)[6] | 50 | 42 (84)[9] |
| Non GBS carriers[5] | 52 | 37 (71; 57–83) | 55 | 24 (44; 30–58)[6] | 107 | 61 (57)[9] |
| Two or more RF | 16 | 16 (100; 79–100) | 23 | 22 (96; 78–99.9) | 39 | 38 (97)[8] |

[1]According to the defined labor ward protocols for each Hospital (see text for details); [2]percentage of women who were eligible for IAP and received it according to protocol; [3]maternal fever defined by hospital protocols (> 37.5°C Hospital A; > 38°C Hospital B); [4]difference between Hospital A and Hospital B not statistically significant ($p = 0.08$); [5]significant difference between Hospital A and Hospital B ($p = 0.01$); [6]difference between GBS carriers and non-carriers with single RF at Hospital B was significant ($p < 0.0001$); [7]difference in compliance between individual RF was highly significant ($p = 0.00001$); [8]difference in compliance between a single RF and two or more was significant ($p = 0.001$); [9]difference in compliance between GBS carriers and non-carriers with single RF was significant ($p = 0.002$); AN, antenatal; PROM, prolongued rupture of membrane; RF, risk factors; GBS, group B streptococcus; IAP, intrapartum antibiotic prophylaxis

GBS carriage, were present in 89 of 198 women (45%; 95% CI, 38–52) with CRF, or 8.1% (95% CI, 6.6–6.9) of all women.

Intrapartum culture results indicated that IAP had been given according to protocol to 89 of 148 women (60%; 95% CI, 52–68) at Hospital A and 84 of 120 (70%; 95% CI, 62–78) at Hospital B who were carrying GBS at delivery. On the other hand, 29 of 96 women (30%; 95% CI, 21–40) at Hospital A and 25 of 83 (30%; 95% CI, 20–41) at Hospital B, who were given IAP because of antenatal GBS carriage alone, were culture negative at delivery.

### Neonatal outcome

Similar proportions of infants at both hospitals (8%) were investigated for sepsis and 94% of them (81 of 86) were treated empirically, with antibiotics. These infants included 49 of 360 (14%; 95% CI, 10–17) whose mothers had been, and 32 of 754 (4.2%; 95% CI, 2.9–5.9) whose mothers had not been, given IAP ($p < 0.001$). Seventeen infants (1.6%) had clinical evidence of sepsis but negative cultures and all recovered without sequelae; the incidence was significantly higher at Hospital B than Hospital A (Table 4). Ten of 17 mothers of infants with clinical sepsis were eligible for IAP and seven had been given it according to protocol.

### DISCUSSION

The proportion of cases of neonatal GBS sepsis that can be prevented by IAP depends on accurate identification of intrapartum GBS carriage and CRF, their respective predictive values and the effectiveness of IAP. Any of several recommended strategies[5] should be cost-effective[14], although none can prevent all cases. In a theoretical comparison, we estimated that strategies A and B would prevent approximately 80 and 70%, respectively, of cases of GBS sepsis and that 20 and 10% of women, respectively, would be eligible for IAP[5]. We rejected a third strategy involving late antenatal screening (at 37 weeks) and IAP for carriers and women with CRF, whose GBS carrier status was unknown, because we estimated that it would involve giving IAP to a very high proportion of healthy women (23.5%). The present study showed that we significantly underestimated the proportions of women who would be eligible for IAP according to any of these strategies.

**Table 4** Infants of women in two cohorts with suspected sepsis

| | *Hospital A* (n = 587) n (%, 95% CI) | *Hospital B* (n = 527) n (%, 95% CI) |
|---|---|---|
| Suspected sepsis | 51 (8.7; 6.5–11.3) | 35 (6.6; 4.7–9.1) |
| Clinically confirmed sepsis[1] | 3 (0.5; 0.104–1.48)[3] | 14 (2.7; 1.28–4)[3] |
| Mothers given/eligible for IAP (indications) | 1/3 (GBS carriage, 1; single RF, 2) | 6/7 (GBS carriage, 4; GBS carriage and RF, 2; single RF, 1) |
| Mother not given IAP (indication if any) | 2 (single RF, 2) | 8 (GBS carriage, 1) |

[1]Clinical hematological or radiological evidence of sepsis and response to antibiotic therapy but negative cultures; [2]95% confidence interval calculated by exact method; [3]difference between Hospital A and B significant ($p = 0.005$, Fisher's exact test); RF, risk factors (see text for details); GBS carriage, positive antenatal vaginal and/or rectal swabs; IAP, intrapartum antibiotic prophylaxis; GBS, group B streptococcus

Maternal GBS carriage is a crude predictor of neonatal sepsis, no matter how accurately it is identified. GBS is part of the normal vaginal flora and fewer than 1% of the infants of carriers will develop sepsis even without IAP. The incidence of GBS carriage in this study was higher (27%) than previously described in Australia[2–4], because we used more sensitive methods to detect it. However, the PPV of antenatal screening before 32 weeks' gestation, for intrapartum GBS carriage, was poor. Added to only fair compliance with protocols, this further limited the benefit of IAP for GBS carriers. A significant proportion of intrapartum carriers did not receive IAP and some who received it were not identified as carriers at the time of delivery. In this study, we included only women whose GBS carrier status was known. In practice compliance with antenatal GBS screening protocols is likely to be only 70–80%[3,15]. This would further reduce the proportion of intrapartum carriers who would receive IAP using this protocol.

Screening at 35–37 weeks' gestation, as recommended in the Centers for Disease Control (CDC) consensus guidelines[6], would predict intrapartum carriage better[16]. This strategy involves administration of IAP to GBS carriers and women with CRF whose culture results are unavailable which, in our population, would be up to 35% of women (GBS carriers 27%; preterm/prescreening deliveries 5%; intrapartum fever 2–3%). An alternative strategy which, until recently, was recommended by the CDC[6] is similar to our protocol B. Based on our study, it would involve giving IAP to about 19% of women in our population (18% with CRF and 1% with history of past GBS infection).

Recently, CDC has published revised guidelines, recommending a single strategy for prevention, based on universal prenatal screening for vaginal and/or rectal GBS colonisation[17]. This revision was based on the results of a retrospective cohort study, which showed a significantly lower rate of perinatal GBS sepsis in infants of women giving IAP on the basis of GBS screening, than in infants of women managed on the basis of risk factors (relative risk, 0.48; 95% CI, 0.37–0.63)[18]. In contrast to our results, the anticipated overall rate of intrapartum antibiotic use, based on the CDC study, was similar for both preventive strategies (31 and 29%, compared with 35 and 19%, respectively, in our study). These differences are apparently due to a higher incidence of risk factors in the USA compared with Australia and failure to account for women given IAP during preterm labour before results of screening are available.

The finding that a strategy based on screening could prevent more cases of GBS sepsis than one based on risk factors alone is not surprising. A risk-factor-based protocol cannot, by definition, prevent sepsis in infants whose mothers have no risk factors. Based on a case–control study of neonatal GBS sepsis, Rosenstein and co-workers estimated that full compliance with a protocol based on late antenatal screening (as above) should prevent 78% of cases of GBS sepsis[9]. By comparison, it was estimated that a protocol based on risk factors alone should prevent a similar proportion of cases in premature infants, in whom most deaths occur, but only 30% of cases in full-term infants, in whom the incidence and mortality are very low[9]. In a more recent study it was estimated that 60% of cases, overall, could be prevented using a risk-factor-based study[19]. A recent review of cases of neonatal GBS septicaemia at Hospital B over an 8-year period showed that a poor outcome correlated with CRF. Four of 20 infants of women with CRF died, whereas all 19 infants of women who had no intrapartum CRF recovered without sequelae[13].

Non-compliance with IAP protocols is often blamed for the occurrence of neonatal GBS sepsis[20], but there has been little evaluation of this claim[21]. Previous studies have shown compliance rates varying from around 80%[10,22] for a protocol based mainly on GBS carriage, to 65% for a CRF-based protocol[11]. However, there has been no previous direct comparison of strategies. In the present study, the rate of compliance with IAP, for women with CRF only, was similar to that for GBS carriers at one hospital, but significantly lower at the other. This probably reflects differences in protocol implementation. At Hospital A, the CRF-based protocol was implemented as part of the study, with specific in-service training of labour ward staff whereas, at Hospital B, a similar protocol had been already implemented when the study began. Poor compliance with IAP for women with CRF at Hospital B was associated with, but probably not the cause of, a significantly higher incidence of clinically diagnosed neonatal sepsis, none of which was shown to be due to GBS. Seven of 14 mothers of affected infants were eligible for IAP and six received it according to protocol.

The fact that compliance rates were similar for both protocols in one hospital suggests that better implementation may have improved compliance with the CRF protocol at the other. Nevertheless, despite the best efforts of the research team, compliance at both hospitals was only fair. This was not due to confusion caused by two protocols being used simultaneously. Chart audits before and after the cohort studies showed that compliance with the CRF-based protocol, at both hospitals, was lower before than during the cohort study. However, despite improvement, especially at Hospital B, it reverted to previously low levels at the end of the cohort study; 65% at Hospital A and 50% at Hospital B[12]. Apparently, the staff at Hospital B were more aware of GBS carriage than CRF as an indication for IAP, despite its poor PPV. This illustrates the importance of staff education, ownership of and responsibility for protocols by unit staff themselves. Changes in the design of record sheets to highlight CRF, ongoing in-service training of staff, standing orders for antibiotic therapy to avoid delays and periodic evaluation of compliance, with feedback to staff, are among strategies we have identified to improve and maintain high rates of compliance.

There is little high-quality evidence[23] to support the use of any IAP strategy. Randomized, controlled trials to compare efficacies of different strategies would require impracticably large sample sizes and use of an untreated control group would not be unethical. Case audits as used in this study, to identify failed prophylaxis or assess protocol compliance, are useful, but not ideal methods of comparison and other factors must also be considered.

The higher proportion of potentially preventable cases of sepsis in full-term infants, when GBS carriage late in pregnancy and selected CRF are the criteria for IAP, compared with CRF only[6,11], must be weighed against the disadvantages of giving antibiotics to nearly twice as many women – as would be indicated in Australia, based on our data[13]. Penicillin anaphylaxis is rare (1–5/10 000)[24], but can be fatal for mother or infant[25] and less severe allergic reactions are common (5–10%). An increase in the proportion of cases of neonatal sepsis due to penicillin/ampicillin-resistant bacteria, associated with increased use of IAP, has been reported[26–29]. In our study, infants of women given IAP were significantly more likely than other infants to be given empirical antibiotic therapy, although few had objective evidence of sepsis. Separation of otherwise normal infants from their mothers and potential complications of intravenous therapy are among the costs of IAP[13,30]. Antibiotic therapy *in utero* or in the first few days of life could affect establishment of the infant's gut flora and ultimately increase the overall prevalence of antibiotic resistance among the normal flora, including GBS[28,31].

Clearly no available strategy for prevention of neonatal GBS sepsis is ideal. IAP can prevent a high proportion of cases if the most sensitive criteria are used but only at the expense of giving antibiotics, unnecessarily, to large numbers of healthy women and their infants. The limited efficiency of IAP is reduced further by poor compliance, unless great attention is paid to implementation and maintenance of protocols. More efficient methods of prevention are needed. Further significant reduction in the incidence of neonatal GBS sepsis is unlikely to be achieved until a conjugate GBS vaccine is available.

### ACKNOWLEDGEMENTS

The authors wish to thank Teresa Leathers, who co-ordinated the study at Hospital A; the midwives and obstetricans at both hospitals for their help and support, particularly Dr Harry Merkur, who was instrumental in initiating the study and Professor Brian Trudinger.

### REFERENCES

1. Boyer KM, Gadzala CA, Kelly PD, Gotoff SP. Selective intrapartum chemoprophylaxis of neonatal group B streptococcal early-onset disease. III: interruption of mother-to-infant transmission. *J Infect Dis* 1983;48:810–16
2. Garland SM, Fliegner JR. Group B streptococcus (GBS) and neonatal infections: the case for intrapartum chemoprophylaxis. *Aust NZ J Obstet Gynaecol* 1991;31:119–22
3. Jeffery HE, McIntosh ED. Antepartum screening and non-selective intrapartum chemoprophylaxis for group B streptococcus. *Aust NZ J Obstet Gynaecol* 1994;34:14–19
4. Vigneswaran R, O'Loughlin JA, McDonald HM. Prevention of early onset group B streptococcal sepsis in the newborn. *J Paediatr Child Health* 1995;31:493–4
5. Gilbert GL, Isaacs D, Burgess MA, *et al.* Prevention of neonatal group B streptococcal sepsis: is routine antenatal screening appropriate? *Aust NZ J Obstet Gynaecol* 1995;35:120–6
6. Schuchat A, Whitney CG, Zangwill KM. Prevention of perinatal group B streptococccal disease: a public health perspective. Centers for Disease Controle and Prevention. *MMWR* 1996; 45(RR-7):1–24
7. Connellan M, Wallace EM. Prevention of perinatal group B streptococcal disease: screening practice in public hospitals in Victoria. *Med J Aust* 2000;172:317–20
8. Isaacs D. Prevention of early onset group B streptococcal infection: screen, treat, or observe? *Arch Dis Child Fet Neonat Ed* 1998;79:F81–F82
9. Rosenstein NE, Schuchat A. Opportunities for prevention of perinatal group B streptococcal disease: a multistate surveillance analysis. The Neonatal Group B Streptococcal Disease Study Group. *Obstet Gynecol* 1997;90:901–6
10. Cheon-Lee E, Amstey MS. Compliance with the Centers for Disease Control and Prevention antenatal culture protocol for preventing group B streptococcal neonatal sepsis. *Am J Obstet Gynecol* 1998;179:77–9
11. Fleming MT, Mc Duffie RS, Russell K, Meikle S. Compliance with risk factor-based guideline for the prevention of neonatal group B streptococcal sepsis. *Infect Dis Obstet Gynecol* 1998;5: 345–8
12. Sanders TR, Roberts CL, Gilbert GL. Compliance with a protocol for intrapartum antibiotic prophylaxis against neonatal group B streptococcal sepsis in women with clinical risk factors. *Infect Dis Obstet Gynecol* 2002;10:223–9
13. Gilbert GL, Hewitt MC, Turner CM, Leeder SR. Epidemiology and predictive values of risk factors for neonatal group B streptococccal sepsis. *Aust NZ J Obstet Gynaecol* 2002;42:497–503
14. Mohle-Boetani JC, Schuchat A, Plikaytis BD, *et al.* Comparison of prevention strategies for neonatal group B streptococcal infection: a population-based economic analysis. *J Am Med Assoc* 1993; 270:1442–8
15. Volumenie JL, Fernandez H, Vial M, *et al.* Neonatal group B streptococcal infection: results of 33 months of universal maternal screening and antibioprophylaxis. *Eur J Obstet Gynecol Reprod Biol* 2001;94:79–85
16. Yancey MK, Schuchat A, Brown LK, *et al.* The accuracy of late antenatal screening cultures in predicting genital group B streptococcal colonization at delivery. *Obstet Gynecol* 1996;88:811–15

17. Schrag SJ, Gorwitz R, Fultz-Butts K, Schuchat A. Prevention of perinatal group B streptococcal diseases. *MMWR* 2002;51(RR11):1–22
18. Schrag SJ, Zell ER, Lynfield R, *et al.* A population-based comparison of straategies to prevent early-onset group B streptococcal disease in neonates. *N Engl J Med* 2002;347:233–9
19. Lin FY, Brenner RA, Johnson YR, *et al.* The effectiveness of risk-based intrapartum chemoprophylaxis for the prevention of early-onset neonatal group B streptococcal disease. *Am J Obstet Gynecol* 2001;184:1204–10
20. Jeffery HE, Moses Lahra M. Eight-year outcome of universal screening and intrapartum antibiotics for maternal group B streptococcal carriers. *Pediatrics* 1998;101:E2
21. Gibbs RS, McDuffie RS, McNabb F, *et al.* Neonatal group B streptococcal sepsis during 2 years of a universal screening program. *Obstet Gynecol* 1994;84:496–500
22. Gervasio CT, Mantaring BS, Alankar S, Shankaran S. Early-onset neonatal group B streptococcal sepsis: intrapartum antibiotic prophylaxis in the clinical setting. *J Perinatol* 2001;21:9–14
23. Smaill F. Intrapartum antibiotics for group B streptococcal colonisation. *Cochrane Database of Systematic Reviews* 2000;CD000115
24. Idsoe O, Guthe T, Willcox RR, Weck AD. Nature and extent of penicillin side-reactions, with particular reference to fatalities from anaphylactic shock. *Bull WHO* 1968;38:159–88
25. Heim K, Alge A, Marth C. Anaphylactic reaction to ampicillin and severe complication in the fetus. *Lancet* 1991;337:859–60
26. Towers CV, Carr MH, Padilla G, Asrat T. Potential consequences of widespread antepartal use of ampicillin. *Am J Obstet Gynecol* 1998;179: 879–83
27. Levine EM, Ghai V, Barton JJ, Strom CM. Intrapartum antibiotic prophylaxis increases the incidence of Gram-negative neonatal sepsis. *Infect Dis Obstet Gynecol* 1999;7:210–13
28. Mercer BM, Carr TL, Beazley DD, *et al.* Antibiotic use in pregnancy and drug-resistant infant sepsis. *Am J Obstet Gynecol* 1999;181:816–21
29. Towers CV, Briggs GG. Antepartum use of antibiotics and early-onset neonatal sepsis: the next 4 years. *Am J Obstet Gynecol* 2002;187:495–500
30. Peralta-Carcelen M, Fargason CA Jr, Cliver SP, *et al.* Impact of maternal group B streptococcal screening on pediatric management in full-term newborns. *Arch Pediatr Adol Med* 1996;150: 802–8
31. Morales WJ, Dickey SS, Bormick P, Lim DV. Change in antibiotic resistance of group B streptococcus: impact on intrapartum management. *Am J Obstet Gynecol* 1999;181:310–14

RECEIVED 07/29/02; ACCEPTED 01/06/03
